# Adherence to the current guidelines on antibiotic prescription among dental practitioners: A national survey

**DOI:** 10.1371/journal.pone.0320528

**Published:** 2025-03-31

**Authors:** Hoda M. Abdellatif, Eman I. Alsagob, Mamata Hebbal, Sree Lalita Kotha, Mohammed Aldossary, Atrey J. Pai Khot, Anu Sara Varghese, Basma Yahya, Ghadah Alajlan, Lamar Alshamrani

**Affiliations:** 1 Public Health Sciences Department, College of Dentistry, Texas A&M University, Dallas, Texas, United States of America; 2 Department of Preventive Dental Sciences, College of Dentistry, Princess Nourah bint Abdulrahman University, Riyadh, Saudi Arabia; 3 Department of Basic Dental Sciences, College of Dentistry, Princess Nourah bint Abdulrahman University, Riyadh, Saudi Arabia; 4 General Directorate of Research and Studies, Ministry of Health, Riyadh, Saudi Arabia; 5 Department of Public Health Dentistry, Goa Dental College and Hospital, Bambolim, Goa, India; 6 Department of Public Health Dentistry, KLE VK Institute of Dental Sciences, KLE Academy of Higher Education and Research, Belagavi, Karnataka, India; 7 Pediatric Resident, Department of Pediatric Dentistry, Ministry of Health Saudi Arabia, Riyadh, Saudi Arabia; 8 Department of Pediatric Dentistry, King Abdullah Bin Abdulaziz University Hospital, Riyadh, Saudi Arabia; 9 Prosthodontics Resident, Department of Prosthodontics, Columbia University College of Dental Medicine, New York, New York, United States of America; Ajman University, UNITED ARAB EMIRATES

## Abstract

**Background:**

The global emergence and spread of antibiotic resistance jeopardise health-care advances and life expectancy. Following the latest antibiotic guidelines is crucial to combat this issue. Therefore, the objective of this study was to assess the knowledge regarding antibiotics prescription and investigate the evidence-based practices among dental practitioners in Saudi Arabia.

**Method:**

This cross-sectional study was conducted during November 2020 to April 2021 in Saudi Arabia. A self-designed validated questionnaire comprising 27 close ended knowledge-based questions and 9 practice-based question was administered among dentists working in various settings through online generated link. A pilot study in 20 dental specialists and consultants was conducted prior to the study, to arise at sample size of 318. Reliability of the questionnaire was assessed with Cronbach’s α value of 0.85, face validity of 84%, and content validity ratio of 0.78. The knowledge and practice score were graded based on quartile derivatives. The data was analyzed using descriptive analysis, chi square analysis, correlation, and regression by IBM SPSS® Statistics for Windows, Version 28.0. Armonk, NY: IBM Corp.

**Results:**

The average age of the participants was 36.3 ±  5.9 years, and their average experience was about 7.3 years. The mean knowledge score was 19.62 ±  4.28 with government employees scoring higher (20.03 ±  3.90) compared to private practitioners and academicians. Clinicians with less than five years of experience had significantly higher knowledge scores (p =  0.002). The majority of the participants, 218 (68.55%), had good practices while 100 (31.44%) participants showed poor practices. Moreover, only half of the participants could correctly identify the majority of case-based scenarios related to antibiotic prescriptions.

**Conclusion:**

Dentists in Kingdom of Saudi Arabia have a good level of knowledge and practices on antibiotics prescription. However, some aspects of poor practices particularly among private sector clinicians necessitate the launching of educational campaigns, interventions and provision of latest guidelines for prudent use of antibiotics in dental practice.

## Introduction

The majority of oral diseases are driven by the oral biofilm, and treatment to abolish oral biofilm is typically provided through various procedures [1]. In certain situations, antibiotics are often used as an adjuvant to active dental therapy to manage acute infection [2]. Antibiotic discovery and use have transformed medical practise and offered public health with tool to control communicable disease [3]. In order to treat or prevent dental infections, dental professionals regularly prescribe antibiotics [4]. Oral diseases that necessitate antibiotic prescriptions are limited to facial cellulitis, acute necrotizing gingivitis, reimplantation of teeth, and trismus [5]. Moreover, patients with medically compromised conditions who run the risk of developing infective endocarditis are advised to take antibiotics [2–4].

Concerns about the emergence of antimicrobial resistance (AMR) due to the overuse of antibiotics, which could lead to a global catastrophe if left unchecked, have been recognized for decades [5,6]. AMR can jeopardise health-care advances and life expectancy [5] and has contributed to an estimated 4.6 million deaths in 2019 positioning it as one of the leading global cause of mortality [5,7]. There is limited information on antibiotic-prescribing practices for prophylaxis and their appropriateness among dental practitioners, despite research in outpatient primary care settings showing that between 30% and 66.5% of antibiotics prescribed are inappropriate [8]. According to a poll of dental professionals, 70% of them said prophylactic antibiotics were administered improperly prior to a dental procedure [9]. The World Health Organization (WHO) has reported alarming global trends of antimicrobial resistance, emphasizing the critical need for antibiotic stewardship [10].

To address the growing threat of AMR, the World Dental Federation has issued international recommendations to dentists and national associations to guide antibiotic prescribing practices in dentistry and mitigate AMR [11]. Similarly, the American Dental Association (ADA) developed and published the latest guidelines for antibiotic usage recommendations in November 2019 offering a comprehensive framework for dentists to make evidence-based decisions on antibiotic prescriptions, ensuring optimal usage while minimizing the risk of resistance [12]. These guidelines are periodically revised to incorporate new evidence and findings from long-term research [12]. The WHO Global Action Plan also identifies dental prescribers as key stakeholders and provides a framework for the development of national action plans (NAPs) to combat AMR [13]. Developed countries including UK and USA have implemented these frameworks through robust prescribing guidelines, educational resources, and practical tools designed for both dental practitioners and patients [14,15].

In contrast, there are no set standards for prescribing antibiotics in developing nations, thus dentists may do so to satisfy their patients’ expectations without offering local therapy [16]. The WHO has identified the Southeast Asia region as the highest-risk area for the emergence and transmission of antibiotic resistance, both in healthcare settings and communities [17]. A similar increase in AMR has been reported in the Eastern Mediterranean region, where programs aimed at curbing AMR are still in the early stages [18]. A study conducted in Saudi Arabia reported antibiotic prescribing methods were extremely problematic [19]. Additionally, the indications and repercussions associated with improper antibiotic prescription have not been demonstrated sufficiently in Saudi Arabia [20]. To fill this research vacuum, this study was conducted to analyze current knowledge and practices of antibiotic usage in dental practice in Saudi Arabia. Previous studies have shown a strong need for activities and training programs that could aid in rationalizing the use of antibiotics by dental professionals [21,22]. Yet, there is an urgent need to explore basis of clinician judgements and to investigate the evidence scale which is translated into clinical practice. Additionally, such study may uncover knowledge gaps amongst both general and specialist dentists in developing countries, offering an opportunity to instruct both dentists and patients. Consequently, the objectives of this survey were to look into dentists’ evidence-based procedures and identify any gaps in their knowledge and practices regarding the prescription of antibiotics. This study utilized clinical scenarios based on the latest antibiotic guidelines to evaluate the current practices of dentists in Saudi Arabia providing a more accurate representation of the real-world decision-making process in dental practice.

## 2. Methodology

### 2.1. Study design and study setting

This cross-sectional study was a research collaboration between Princess Nourah bint Abdulrahman University (PNU) and the Ministry of Health (MOH) conducted to assess the current knowledge and practice of antibiotic use in Saudi Arabia in accordance with the latest antibiotics’ guidelines [23]. Dentists working in various settings such as educational institutions, hospitals, and dental centers from all regions of Saudi Arabia were the target population. Undergraduate dental students were excluded from the study. The study was conducted during November 2020 to April 2021.

### 2.2. Ethical considerations

The Princess Nourah bint Abdulrahman University Institutional Review Board (IRB) granted their approval for the study’s ethical conduct (Ref. no 20-0320), and it was then carried out in compliance with STROBE regulations. Additional information regarding the ethical, cultural, and scientific considerations specific to inclusivity in global research is included in the Supporting Information (Supplementary file: S1 Checklist). Furthermore, it is verifiable that the study was carried out in complete line with the Declaration of Helsinki of the World Medical Association and the ethical standards of the Saudi Ministry of Health.

### 2.3. Drafting and elements of the questionnaire

To gauge participants’ current understanding and use of antibiotics, a self-designed questionnaire was developed using Google forms. The questionnaire was modified and adopted from previous studies [24–27]. Prior to this investigation, a pilot study with 20 dental consultants and specialists validated this questionnaire. The knowledge segment’s reliability using Cronbach’s alpha value was determined to be 0.871 and the attitude segment’s reliability was found to be 0.844, indicating that the questionnaire was well-structured and appropriate for the target demographic. Subject matter experts thoroughly analyzed and evaluated the questionnaire’s face (83%) and content validity (0.81) for readability, clarity, and comprehensiveness of the questions. The participants were sent a link to the questionnaire and a consent form using email as a platform. There was no enticement or hint offered to participants during the filling of questionnaire. Discretion of information acquired was secured during the study. It had four sections with close ended multiple-choice questions. The first section (Section A) consisted of questions about participants’ demographic data, including gender, age, clinical title, education, clinical experience and work place related points. The second section (Section B) assessed the participants’ current knowledge of antibiotic prescription guidelines for patients with various medical and dental conditions comprised of 27 questions. The third section (Section C) riveted participants’ reflections on their antibiotic prescription practice which included 9 questions and 10 case scenarios encountered in clinical dental practice. The questionnaire has been added as supporting information (Supplementary S1 File).

### 2.4. Sample size and sampling technique

The sample size was estimated to be 307 using the formula n =  4pq/d^2^, based on the pilot study (p =  55.6%), where p =  prevalence, q =  1-p, and d =  allowable error (10% of p), α =  0.05, β =  0.2, arising at a final sample size of 318. A convenient sampling technique was employed for data collection. For the governmental sector, a database of dentists from the MOH in Saudi Arabia was obtained, while for the private sector and academic professionals, a list of faculty members was sourced from universities and specialty associations. According to the SCFHS Health Care Report 2017, there are 19,239 registered dentists (including dentists and specialists) in Saudi Arabia [28]. Emails were sent to all participants, explaining the purpose of the research along with the questionnaire and written informed consent form. Three reminder emails were sent at two-week intervals in case the completed forms were not received.

### 2.5. Data collection and scoring criteria

An introductory message described the study aim and the participants’ voluntary involvement. Using Google Forms (a free web-based survey generator), the questionnaire was transformed into an electronic form and the link was distributed to the Saudi dentist community via the Ministry of Health (MOH). Participants were asked to complete the questionnaire, which was in English, the primary language of instruction in Saudi Arabia, within two to three weeks of receiving it. Two reminders were sent at one-week intervals to encourage completion. A final reminder was sent three weeks after the initial invitation. Any forms submitted after the deadline were excluded from the analysis. No personal information was collected during the study, and a note reminding participants to maintain discretion was included in the questionnaire. The grading system employed for the survey based on quartile derivatives [29]. The knowledge and practice score were computed by assigning one point for each accurate or positive response, and each incorrect or negative response received zero points. The final scores were given as a percentage after summing up each participant’s points and calculating the percentages. the determined knowledge score was classified into three categories: poor knowledge (0–40%), fair knowledge (41 < 70%) and good knowledge (70% and above). Similarly, practice score was categorized into poor practice ( ≤ 50) and good practice (>60%). The maximum possible cumulative score in Section B (knowledge) was 27, while that in Section C (practice) was 9.

### 2.6. Statistical analysis

Excel sheet was generated from google form and data was analyzed using IBM Corp. Released 2021. IBM SPSS® Statistics for Windows, Version 28.0. Armonk, NY: IBM Corp. Descriptive statistics like frequencies, percentages, mean, and standard deviation of the dental practitioners were calculated. Subsequently, Chi-square test, Mann–Whitney U test and Kruskal–Wallis test were performed to find differences between knowledge, practice scores. In addition, the correlation between the knowledge and practice scores was evaluated by Spearman’s rank correlation coefficient test whereas, their association with the demographic details of the dental practitioners was analyzed by simple linear regression and multivariate linear regression analysis. A *p*-value of <  0.05 was considered statistically significant.

## 3. Results

### 3.1. Sociodemographic characteristics of participants

A total of 318 (39.9% male and 60.1% female) participants returned completed questionnaires. The majority 123 (38.8%) of the participants were specialist, 88 (27.8%) were consultants, 57 (18.0%) were interns, 38 (12.0%) were general dentist and least were resident 11 (3.5%) working in health sector. The average age of the participants was 36.3 ±  5.9 years, and their average experience was about 7.3 years. The majority of participants were belonging to Riyadh province (38.7%) and government sector (67.9%). The demographic and professional characteristics of participants are shown in Table 1.

**Table 1:** Sociodemographic characteristics of dentists participating in survey.

Characteristics		N (%)
Gender	Female	191 (60.1)
	Male	127 (39.9)
Age group	25–35	184 (57.9)
	36–45	76 (23.9)
	46–55	48 (15.1)
	>55	10 (3.1)
Practice sector	Private sector clinician	22 (6.9)
	Government sector clinician	216 (67.9)
	Academician	8 (2.5)
	Both	72 (22.6)
Clinical title	Consultant	88 (27.8%)
	Specialist	123 (38.8%)
	Resident	11 (3.5%)
	General dentist	95 (29.87%)
Clinical experience	< 5 years	161 (50.6)
	5–10 years	45 (14.2)
	11–15 years	53 (18.6)
	>15 years	59 (18.6)
Workplace	Al- Jouf province	10 (3.1)
	Al-Bahah province	40 (12.6)
	Asir province	13 (4.1)
	Eastern province	44 (13.8)
	Hail province	7 (2.2)
	Jazan province	12 (3.8)
	Madinah province	30 (9.4)
	Makkah province	11 (3.5)
	Najran province	1 (0.3)
	Northern border	18 (5.7)
	Qassim province	4 (1.3)
	Riyadh province	123 (38.7)
	Tabuk province	5 (1.6)
Undergraduate training	Saudi Arabia	275 (86.5%)
	Europe	5 (1.6%)
	North America	10 (3.1%)
	Asian Countries	12 (3.8%)
	Others	16 (5%)
Postgraduate training	Saudi Arabia	176 (55.3%)
	Europe	16 (5%)
	North America	16 (5%)
	Asian Countries	2 (0.6%)
	Egypt	7 (2.2%)
	Others	101 931.8%)

All values are expressed as frequency with percentages (in parentheses).

### 3.2. Knowledge among dentists regarding indications for antibiotic prescription

Table 2 shows distribution of study participants according to knowledge of antibiotic prescription with relevant clinical situation according to clinical practice sector. The analysis showed that correct pattern of prescribing antibiotics was statistically significant for dental diseases such as pulp necrosis (*p* =  0.06), apical periodontitis (*p* =  0.003), draining sinus (*p* =  0.005), abscess (*p* =  ≤  0.001), and fracture of teeth (*p* =  ≤  0.001). The prescription of antibiotics was indicated in surgical dental procedures which was found to be significantly higher in government sector clinician as compared to private sector and academician (*p* ≤  0.05). Similarly, majority of the participants were aware regarding antibiotics prescription in a high-risk medical condition such as infective endocarditis (*p* =  0.026), congenital cardiac abnormalities (*p* =  0.041), and prosthetic cardiac valves (*p* =  0.004).

**Table 2:** Knowledge among dentists regarding indications for antibiotic prescription according to clinical practice sector.

Knowledge based Questions	Response	Practice sector (N=318)				*p*-Value
		Private sector clinician*n* (%)	Government sector clinician*n* (%)	Academician*n* (%)	Both*n* (%)	
**Dental diseases**						
Reversible pulpitis	Yes^‡^	2 (9.1)	11 (5.1)	1 (12.5)	1 (1.4)	0.372
	No^¶^	19 (86.4)	203 (94.0)	7 (87.5)	69 (95.8)	
	Don’t know^‡^	1 (4.5)	2 (0.9)	0	2 (2.8)	
Irreversible pulpitis	Yes^‡^	4 (18.2)	25 (11.6)	0	5 (6.9)	0.1
	No^¶^	15 (68.2)	185 (85.6)	8 (100)	63 (87.5)	
	Don’t know^‡^	3 (13.6)	6 (2.8)	0	4 (5.6)	
Pulp necrosis	Yes^‡^	4 (18.2)	21 (6.60)	0	3 (4.2)	.006^*^
	No^¶^	13 (59.1)	185 (58.18)	8 (100)	64 (88.9)	
	Don’t know^‡^	5 (22.7)	10 (3.14)	0	5 (6.9)	
Apical periodontitis	Yes^‡^	8 (36.4)	38 (17.6)	0	10 (13.9)	.003^*^
	No^¶^	10 (45.5)	168 (77.8)	6 (75)	58 (80.6)	
	Don’t know^‡^	4 (18.2)	10 (4.6)	2 (25)	4 (5.6)	
Draining dental sinus tract	Yes^‡^	4 (18.2)	53 (24.5)	2 (25)	16 (22.2)	.005^*^
	No^¶^	14 (63.6)	160 (74.1)	6 (75)	52 (72.2)	
	Don’t know^‡^	4 (18.2)	3 (1.4)	0	4 (5.6)	
Localized intraoral abscess	Yes^‡^	7 (31.8)	64 (29.6)	3 (37.5)	30 (41.7)	≤ 0.001^**^
	No^¶^	9 (40.9)	143 (66.2)	5 (62.5)	40 (55.6)	
	Don’t know^‡^	6 (27.3)	9 (4.2)	0	2 (2.8)	
Cellulitis	Yes^¶^	18 (81.8)	202 (93.5)	8 (100)	71 (98.6)	0.159
	No^‡^	3 (13.6)	10 (4.6)	0	1 (1.4)	
	Don’t know^‡^	1 (4.5)	4 (1.9)	0	0	
Acute ulcerative gingivitis	Yes^‡^	16 (72.7)	122 (56.5)	5 (62.5)	46 (63.9)	0.314
	No^¶^	3 (13.6)	80 (37)	3 (37.5)	22 (30.6)	
	Don’t know^‡^	3 (13.6)	14 (6.5)	0	4 (5.6)	
Chronic marginal gingivitis	Yes^‡^	1 (4.5)	18 (8.3)	0	6 (8.3)	0.054
	No^¶^	14 (63.6)	178 (82.4)	6 (75)	59 (81.9)	
	Don’t know^‡^	7 (31.8)	20 (9.3)	2 (25)	7 (9.7)	
Aggressive periodontitis	Yes^¶^	15 (68.2)	142 (65.7)	4 (50)	43 (59.7)	0.798
	No^‡^	4 (18.2)	55 (25.5)	3 (37.5)	23 (31.9)	
	Don’t know^‡^	3 (13.6)	19 (8.8)	1 (12.5)	6 (8.3)	
Moderate periodontitis	Yes^‡^	3 (13.6)	14 (6.5)	0	6 (8.3)	0.562
	No^¶^	18 (81.8)	183 (84.7)	8 (100)	63 (87.5)	
	Don’t know^‡^	1 (4.5)	19 (8.8)	0	3 (4.2)	
Mild pericoronitis	Yes^‡^	0	14 (6.5)	2 (25)	6 (8.3)	0.094
	No^¶^	19 (86.4)	193 (89.4)	6 (75)	64 (88.9)	
	Don’t know^‡^	3 (13.6)	9 (4.2)	0	2 (2.8)	
Dry socket	Yes^‡^	7 (31.8)	58 (26.9)	2 (25)	23 (31.9)	0.215
	No^¶^	10 (45.5)	141 (65.3)	6 (75)	44 (61.1)	
	Don’t know^‡^	5 (22.7)	17 (7.9)	0	5 (6.9)	
Fracture of tooth	Yes^‡^	7 (31.8)	13 (6)	1 (12.5)	8 (11.1)	≤ 0.001^**^
	No^¶^	10 (45.5)	189 (87.5)	7 (87.5)	52 (72.2)	
	Don’t know^‡^	5 (22.7)	14 (6.5)	0	12 (16.7)	
**Dental procedures**						
Scaling and Root planning	Yes^‡^	0	8 (3.7)	0	2 (2.8)	≤ 0.001^**^
	No^¶^	20 (90.9)	208 (96.3)	8 (100)	70 (97.2)	
	Don’t know^‡^	2 (9.1)	0	0	0	
Simple extraction	Yes^‡^	1 (4.5)	4 (1.9)	0	2 (2.8)	.024^*^
	No^¶^	20 (90.9)	212 (98.1)	8 (100)	70 (97.2)	
	Don’t know^‡^	1 (4.5)	0	0	0	
Surgical extraction	Yes^¶^	7 (31.8)	79 (36.6)	4 (50)	33 (45.8)	0.278
	No^‡^	12 (54.5)	128 (59.3)	4 (50)	37 (51.4)	
	Don’t know^‡^	3 (13.6)	9 (4.2)	0	2 (2.8)	
Root canal treatment	Yes^‡^	3 (13.6)	4 (1.9)	0	5 (6.9)	≤ 0.001^**^
	No^¶^	14 (63.6)	206 (95.4)	8 (100)	63 (87.5)	
	Don’t know^‡^	5 (22.7)	6 (2.8)	0	4 (5.6)	
Apicectomy	Yes^‡^	11 (50)	86 (39.8)	5 (62.5)	37 (51.4)	0.285
	No^¶^	8 (36.4)	100 (46.3)	3 (37.5)	31 (43.1)	
	Don’t know^‡^	3 (13.6)	30 (13.9)	0	4 (5.6)	
Routine local anaesthesia injections	Yes^‡^	8 (36.4)	55 (25.5)	4 (50)	25 (34.7)	0.173
	No^¶^	13 (59.1)	142 (65.7)	4 (50)	46 (63.9)	
	Don’t know^‡^	1 (4.5)	19 (8.8)	0	1 (1.4)	
Restorative procedures	Yes^‡^	4 (18.2)	26 (12)	3 (37.5)	21 (29.2)	.002^*^
	No^¶^	14 (63.6)	179 (82.9)	5 (62.5)	45 (62.5)	
	Don’t know^‡^	4 (18.2)	11 (5.1)	0	6 (8.3)	
Dental implant placement	Yes^¶^	11 (50)	94 (43.5)	1 (12.5)	38 (52.8)	0.179
	No^‡^	8 (36.4)	97 (44.9)	7 (87.5)	26 (36.1)	
	Don’t know^‡^	3 (13.6)	25 (11.6)	0	8 (11.1)	
**Medical conditions**						
Cardiac pacemakers	Yes^‡^	11 (50)	105 (48.6)	6 (75)	28 (38.9)	0.327
	No^¶^	6 (27.3)	81 (37.5)	2 (25)	33 (45.8)	
	Don’t know^‡^	5 (22.7)	30 (13.9)	0	11 (15.3)	
Congenital cardiac abnormalities	Yes^¶^	14 (63.6)	131 (60.6)	7 (87.5)	34 (47.2)	.041^*^
	No^‡^	3 (13.6)	66 (30.6)	1 (12.5)	26 (36.1)	
	Don’t know^‡^	5 (22.7)	19 (8.8)	0	12 (16.7)	
Previous infective endocarditis	Yes^¶^	19 (86.4)	203 (94)	8 (100)	71 (98.6)	.026^*^
	No^‡^	2 (9.1)	6 (2.8)	0	0	
	Don’t know^‡^	1 (4.5)	7 (3.2)	0	1 (1.4)	
Prosthetic cardiac valves	Yes^¶^	14 (63.6)	196 (90.7)	7 (87.5)	64 (88.9)	.004^*^
	No^‡^	3 (13.6)	8 (3.7)	1 (12.5)	6 (8.3)	
	Don’t know^‡^	5 (22.7)	12 (5.6)	0	2 (2.8)	
Prosthetic joints	Yes^‡^	6 (27.3)	79 (36.6)	5 (62.5)	28 (38.9)	0.373
	No^¶^	8 (36.4)	94 (43.5)	2 (25)	32 (44.4)	
	Don’t know^‡^	8 (36.4)	43 (19.9)	1 (12.5)	12 (16.7)	

All values are expressed as the frequency with percentages (in parentheses).

¶ denotes correct response, and

‡ denotes wrong response. Statistical test used: chi-square test. Level of significance:

* *p* ≤  0.05 is considered statistically significant,

** *p* ≤  0.001 Highly significant association.

### 3.3. Practice of antibiotic prescription among dentists according to clinical practice sector

Table 3 depict trends in dental antibiotic prescription practices according to clinical guidelines. The antibiotics were always prescribed for demanding clinical conditions in which they are appropriate (90.3%) with statistically significance difference between clinical practice sector (*p* =  0.043) and the possible consequences of non-adherence to therapy (*p* = 0.001). About 45% of the private clinician said they would prescribe antibiotics to defer the treatment, in case of long waiting queues with statistically insignificant difference between clinical practice sector (*p* =  0.281). About 97.2% of the government clinician were aware of antimicrobial resistance which deferred them from prescribing antibiotics without confirmed diagnosis (*p* =  0.039) and past history of antibiotics intake (*p* =  0.016). Most of the clinician (58%) preferred to use amoxicillin to treat their patients. The clinician agreed upon Clindamycin being the drug of choice for patients who were allergic to penicillin (*p* =  0.006). Nearly, 90.3% of the participants follow AHA guidelines for antibiotic prophylaxis and 47.2% read journal articles to learn about antibiotics prescription. Two thirds (68.77%) of participants were aware of available guidelines/recommendations for prescribing antibiotics in dentistry, and they had last updated their knowledge within the past 2 years.

**Table 3:** Practice of antibiotic prescription among dentists according to clinical practice sector.

Practice based Questions	Response	Practice sector				*p*-Value
		Private sector clinician *n* (%)	Government sector clinician *n* (%)	Academician *n* (%)	Both *n* (%)	
If clinical condition demands antibiotic prescription	Always^¶^	18 (81.8)	155 (71.8)	6 (75)	65 (90.3)	.043^*^
	Never^‡^	0	11 (5.1)	1 (12.5)	2 (2.8)	
	Occasion^‡^	4 (18.2)	50 (23.1)	1 (12.5)	5 (6.9)	
If patient requests antibiotic prescription	Always^‡^	1 (4.5)	8 (3.7)	1 (12.5)	5 (6.9)	0.39
	Never^¶^	17 (77.3)	188 (87)	7 (87.5)	63 (87.5)	
	Occasion^‡^	4 (18.2)	20 (9.3)	0	4 (5.6)	
Prescribing antibiotics, if you are uncertain of diagnosis	Always^‡^	0	7 (3.2)	1 (12.5)	3 (4.2)	.039^*^
	Never^¶^	10 (45.5)	154 (71.3)	5 (62.5)	55 (76.4)	
	Occasion^‡^	12 (54.5)	55 (25.5)	2 (25)	14 (19.4)	
Prescribing antibiotics, to sustain patients, until specialist is available	Always^‡^	2 (9.1)	7 (3.2)	0	4 (5.6)	0.18^*^
	Never^¶^	8 (36.4)	160 (74.1)	6 (75)	53 (73.6)	
	Occasion^‡^	12 (54.5)	49 (22.7)	2 (25)	15 (20.8)	
To defer the treatment, in case of long waiting queues	Always^‡^	1 (4.5)	9 (4.2)	0	3 (4.2)	0.281
	Never^¶^	11 (50)	154 (71.3)	7 (87.5)	55 (76.4)	
	Occasion^‡^	10 (45.5)	53 (24.5)	1 (12.5)	14 (19.4)	
Are you aware of “antimicrobial resistance	Yes^¶^	21 (95.5)	210 (97.2)	8 (100)	72 (100)	0.441
	No^‡^	1 (4.5)	6 (2.8)	0	0	
Do you inquire from your patient about past history of antibiotics in the past 1 week, before prescribing antibiotics?	Yes^¶^	8 (36.4)	159 (73.6)	5 (62.5)	52 (72.2)	.016^*^
	No^‡^	4 (18.2)	13 (6)	0	6 (8.3)	
	Occasionally^‡^	10 (45.5)	44 (20.4)	3 (37.5)	14 (19.4)	
Do you advise your patient to adhere to the dosage regimen and inform the consequences	Yes^¶^	10 (45.5)	172 (79.6)	6 (75)	55 (76.4)	.001^*^
	No^‡^	3 (13.6)	2 (0.9)	1 (12.5)	4 (5.6)	
	Occasionally^‡^	9 (40.9)	42 (19.4)	1 (12.5)	13 (18.1)	
Which antibiotic(s) do you most often prescribe therapeutically, for your patients?	Amoxicillin	12 (54.5)	127 (58.8)	3 (37.5)	42 (58.3)	0.891
	Amoxicillin & Clavulanic acid	6 (27.3)	60 (27.8)	4 (50)	19 (26.4)	
	Amoxicillin & Metronidazole	2 (9.1)	18 (8.3)	1 (12.5)	9 (12.5)	
	Clindamycin	0	2 (0.9)	0	2 (2.8)	
	Azithromycin	0	2 (0.9)	0	0	
	Ofloxacin	0	1 (0.5)	0	0	
	Metronidazole	2 (9.1)	5 (2.3)	0	0	
	Others	0	1 (0.5)	0	0	
Which antibiotic do you prescribe for your patients, allergic to penicillin?	Erythromycin	7 (31.8)	55 (25.5)	2 (25)	17 (23.6)	.006^*^
	Azithromycin	1 (4.5)	34 (15.7)	0	5 (6.9)	
	Clindamycin	10 (45.5)	111 (51.4)	5 (62.5)	45 (62.5)	
	Clarithromycin	1 (4.5)	2 (0.9)	0	0	
	Cephalosporin	3 (13.6)	13 (6)	0	5 (6.9)	
	Others	0	1 (0.5)	1 (12.5)	0	
Do you follow current guidelines for antibiotic prophylaxis?	Always^¶^	9 (40.9)	156 (72.2)	6 (75)	50 (69.4)	.016^*^
	Never^‡^	3 (13.6)	12 (5.6)	0	0	
	Occasionally^‡^	10 (45.5)	48 (22.2)	2 (25)	22 (30.6)	
If yes, which guidelines do you follow?	AHA guidelines	18 (81.8)	196 (90.7)	8 (100)	65 (90.3)	0.485
	NICE guidelines	2 (9.1)	15 (6.9)	0	6 (8.3)	
	Others	2 (9.1)	5 (2.3)	0	1 (1.4)	
What was your main source of updating your knowledge regarding antibiotic prescription guidelines?	Textbooks	11 (50)	39 (18.1)	4 (50)	20 (27.8)	.016^*^
	Conferences	0	8 (3.7)	0	1 (1.4)	
	Journal articles	6 (27.3)	106 (49.1)	2 (25)	34 (47.2)	
	Continuing education courses	1 (4.5)	34 (15.7)	2 (25)	13 (18.1)	
	Colleagues	4 (18.2)	22 (10.2)	0	1 (1.4)	
	Others	0	7 (3.2)	0	3 (4.2)	
When did you last update your knowledge regarding antibiotic prescription guidelines?	Within the last two years^¶^	13 (59.1)	162 (75)	5 (62.5)	40 (55.6)	0.061
	Within the last five years^‡^	8 (36.4)	46 (21.3)	3 (37.5)	29 (40.3)	
	Others^‡^	1 (4.5)	8 (3.7)	0	3 (4.2)	

All values are expressed as the frequency with percentages (in parentheses).

¶ denotes good practice, and

‡ denotes poor practice. Statistical test used: chi-square test. Level of significance:

* *p* ≤  0.05 is considered statistically significant,

** *p* ≤  0.001 Highly significant association.

### 3.4. *Knowledge and Practice scores regarding antibiotic prescriptions among dentists*

**Fig 1:**
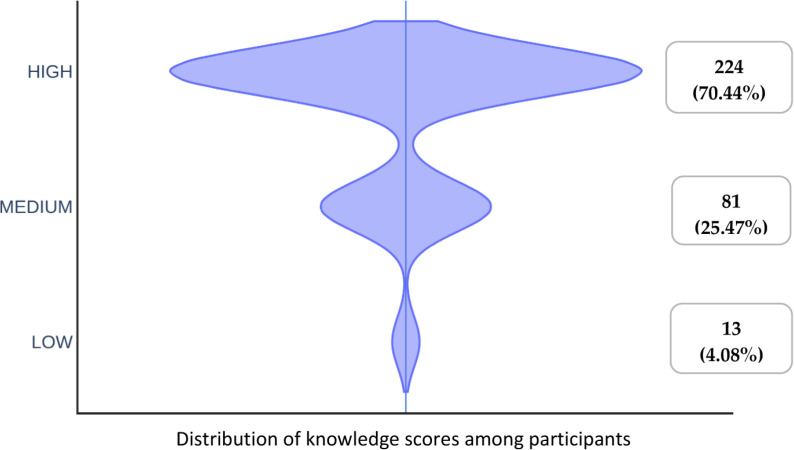
Violin plot depicting distribution of knowledge scores among the participants.

**Fig 2:**
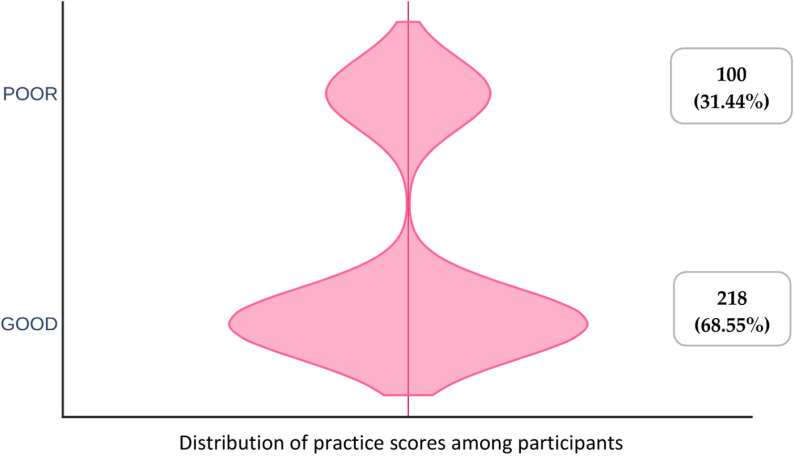
Violin plot depicting distribution of practice scores among the participants.

Regarding antibiotic prescriptions for clinical signs, the mean knowledge score was 19.62 ±  4.28. Based on the quartile derivative, knowledge score was graded as high, medium and low. Kruskal–Wallis test depicted that there was higher significant knowledge score (*p* =  0.04) between the participants with extreme age groups (25-35, > 55). It was evident that participants employed in government sector had higher knowledge score (20.03 ±  3.90). The clinical experience influences the knowledge score significantly wherein recently graduated clinician (5 years) was found to had higher knowledge score which was statistically significant (*p* =  0.002). However, the majority of participants, 224 (70.44%), had high knowledge score, whereas 81 (25.47%) had medium and 13 (4.08%) had low knowledge scores (Fig 1). There was significant difference in the practice scores between the participants with age (25-35, 36-45, 46-55, > 55), practice wise distribution (private, government, academics and both) and clinical experience (,5, 5-10, 11-15, > 15 years by Kruskal–Wallis test (*P* <  0.05). The two third of the participants, 218 (68.55%), had good practices and, on the other hand, 100 (31.44%) participants had a poor practice towards the antibiotic prescription (Fig 2). Mann–Whitney U test showed that there was significant difference in the knowledge and practice scores between the participants by gender wise distribution (*P* ≤  0.001) (Table 4).

**Table 4:** Knowledge and Practice scores regarding antibiotic prescriptions among dentists.

Characteristics		Knowledge score Mean ± SD	*p*-Value	Practice score Mean ± SD	*p*-Value
Gender ^α^	Female	20.07 ± 4.34	< 0.001^**^	8.04 ± 1.16	.001^*^
	Male	18.81 ± 4.09		7.52 ± 1.39	
Age group^β^	25-35	20.23 ± 4.16	.004^*^	8.08 ± 1.14	< 0.001^**^
	36-45	18.87 ± 4.53		7.33 ± 1.42	
	46-55	18.63 ± 4.00		7.67 ± 1.29	
	>55	20.30 ± 4.08		8.00 ± 1.49	
Practice sector ^β^	Private sector clinician	16.36 ± 6.64	.078	6.77 ± 1.77	.014^*^
	Government sector clinician	20.03 ± 3.90		7.98 ± 1.16	
	Academics only	19.36 ± 4.27		7.75 ± 0.89	
	Clinics and Academics	19.42 ± 4.10		7.74 ± 1.33	
Clinical title ^β^	Consultant	19.66 ± 3.93	.193	7.76 ± 1.22	.359
	Specialist	18.95 ± 4.16		7.61 ± 1.46	
	Resident	18.89 ± 4.94		7.77 ± 1.49	
	General dentist	20.12 ± 4.26		7.99 ± 1.15	
Clinical experience ^β^	< 5 years	20.37 ± 4.14	.002^*^	8.06 ± 1.12	.008^*^
	5-10 years	18.07 ± 4.98		7.71 ± 1.44	
	11-15 years	18.88 ± 4.02		7.32 ± 1.46	
	>15 years	19.41 ± 3.92		7.78 ± 1.25	

All values are expressed as mean ±  standard deviation (SD). The statistical test used:

α Mann-Whitney U test,

β Kruskal-Wallis test; Level of significance

* *p* ≤  0.05 is considered statistically significant,

** *p* ≤  0.001 Highly significant association.

### 3.5. Relationship between variables using Spearman’s rank correlation coefficient test and multivariant regression analysis

A positive linear correlation (r =  + .491) and a high statistically significant difference (*P ≤  0.001*) between the knowledge and practice scores among the participants was found by the Spearman’s rank correlation coefficient test. The knowledge had been empowered to be negatively correlated with undergraduate training (*p* =  0.003) and positively correlated with postgraduation training (*p* =  0.061). However, Practice was observed to be significantly correlated with postgraduate training (*p* =  0.012) (Table 5). The simple linear regression analyses did not show any statistically significant difference between the knowledge and practice of correct prescription and socio-demographic characteristics. Multiple linear regression analysis revealed that the better knowledge and practice scores were significantly associated with gender (*P* ≤  0.05) and not with age, clinical title, practice sector and clinical experience with dependence value of Adjusted R Square =  0.011, 0.058 respectively (Table 6).

**Table 5:** Correlation of knowledge and practice scores regarding antibiotic prescriptions among dentists.

Parameters		Undergraduate training	Postgraduate training
Knowledge^¥^	*r*	-0.162^**^	0.105
	*p*	0.003^*^	0.063
Practice^€^	*r*	-0.016	.140^*^
	*p*	0.777	0.012^*^

The statistical test used: Spearman’s rank correlation coefficient test; Level of significance:

¥ and

€ have highly statistically significant correlation.

* *P* ≤  0.05 is considered a statistically significant correlation.

**Table 6:** Relationship between sociodemographic characteristics and knowledge/practices scores regarding antibiotic prescriptions among dentists.

Parameters	Coefficient (r)	SE	t	95% CI		*p*-Value	Adjusted R^2^
				Lower	Upper		
	**Dependent variable: Knowledge score**						
(Constant)	–	1.030	17.150	15.636	19.688	–	.011
Gender	.134	.502	2.369	.199	2.143	.018*	
Age	.028	.259	.431	-.398	.620	.667	
Clinical title	.055	.196	.838	-.224	.460	.556	
Practice sector	.071	.283	1.229	-.209	.904	.220	
Clinical Experience	.039	.257	.452	-.387	.792	.618	
	**Dependent variable: Practice score**						
(Constant)	–	.300	24.220	6.684	7.866	–	.058
Gender	.194	.144	3.504	.221	.789	.001^*^	
Age	.167	.075	2.685	.054	.351	.058	
Clinical title	.020	.058	.271	-.098	.129	.787	
Practice sector	.086	.082	1.530	-.036	.288	.127	
Clinical Experience	.128	.074	1.589	-.265	.028	.113	

CI: confidence interval; SE: Standard error; The statistical test used: Multivariate linear regression model; Level of significance:

* *p* ≤  0.05 is considered statistically significant.

### 3.6. Case based scenario evaluation among the participants

A vast majority of dentists, i.e., 46.8%, did not routinely prescribe broad-spectrum antibiotics in contrast to 31.1% of dentists that did so while 22.1% of dentists only used them depending on the case. Table 7 shows the responses of the participants about antibiotic prescriptions, broken down by the percentage of participants for each clinical scenario. More than half of the participants correctly identified the clinical cases. It was seen that 85.6% of dentists interviewed followed standard protocols for duration and dosage for antibiotics while 10% would prescribe these medicines on a case-to-case basis.

**Table 7:** Participating Saudi dentists’ responses regarding antibiotics prescription by percentage of respondents to each item.

Item (Case)	Correct Answer	Correct Answer N (%)	Wrong Answer N (%)	I don’t know N (%)
Case 1: You have just performed a surgical extraction of grossly decayed #46, in a known diabetic patient whose random blood glucose level was 180 mg/dl. The surgery lasted about one hour long.	Yes	178 (56%)	92 (28%)	48 (15%)
Case 2: In ER, a parent reported that one of her 9-year old child’s teeth was extremely painful. The child has no fever. However, an intraoral swelling was found in relation to #74, along with halitosis and discharging pus.	No	160(50.3%)	130(40.9%)	28 (8.8%)
Case 3: A 6-year old girl reported to the ER with a history of fall, while skating on the road. On clinical examination, her upper lip was edematous and the anterior maxillary alveolar segment was mobile.	Yes	195(61.3%)	77(24.2%)	46(14.5%)
Case 4: You have planned to perform scaling and root planing in a 20-year old male patient who was already diagnosed with a genetic disorder, Marfan syndrome who had undergone repair of mitral valve 2 years ago.	Yes	151(47.5%)	83(26.1%)	84(26.4%)
Case 5: A 23-year old patient presented with bad breath, restricted mouth opening and pain in relation to the partially erupted lower third molar. The area around the tooth was erythematous and swollen.	Yes	200(62.9%)	95(29.9%)	23(7.2%)
Case 6: You noticed a vertical fracture in a painful right upper first molar extending to the furcation, and made a treatment plan of extraction. However, the patient is unwilling to undergo extraction.	No	259(81.4%)	31(9.7%)	28(8.8%)
Case 7: A 65-year old patient with chronic kidney disease, undergoing hemodialysis twice every week needs an endodontic treatment of #46, Tooth is associated with a periapical abscess.	Yes	136(42.8%)	111(34.9%)	71(22.3%)
Case 8: You advise a female patient to come for periodontal surgery after two days. Her medical history is unremarkable, except that she is a carrier of sickle cell anemia.	No	151(47.5%)	83(26%)	84(26.4%)
Case 9: You need to perform incision and drainage of a submandibular swelling in a patient with a history of coronary artery bypass grafting six months ago. Her general physical status is currently stable.	Yes	193(60.7%)	73(23%)	52(16.4%)
Case 10: An apprehensive parent, reports that her daughter had multiple ulcers of lower lip from the time orthodontic bonding done a week ago, as they would be out of town for ten days, she insists for an antibiotic prescription for her daughter to prevent infection.	No	266(83.6%)	26(8.2%)	26(8.2%)

All values are expressed as frequency with percentages (in parentheses).

## 4. Discussion

Over the years, Saudi Arabia has witnessed a significant surge in antimicrobial resistance, which has been worsened by the unauthorized use of antibiotics, inappropriate prescribing practices by healthcare professionals, and self-administration of antibiotics by patients [30]. Because of the serious implications for public health, the proper use of antibiotics has emerged as a critical issue on the health-care agenda. Clinicians’ antimicrobial prescribing practices and knowledge should be assessed in order to design future interventions to ensure rational antimicrobial use and reduce the risk of antimicrobial resistance [31]. This study is the first of its kind to assess dentists’ antibiotic prescription knowledge and practices in Saudi Arabia through presenting various clinical scenarios and correlating knowledge with antibiotic prescription practices according to the most recent updated guidelines. The findings of this study revealed that dentists’ prescription patterns aligned with the updated antibiotic guidelines.

### 4.1. Guidelines adherence

In the current study, majority of the dentists recommended against using antibiotics for dental diseases such as irreversible pulpitis, pulp necrosis, apical periodontitis, draining sinus, abscess, and tooth fracture. This was in contrast to other studies that found a significant amount of antibiotics being used for inappropriate therapeutic indications [31,32]. Recent research and guidelines emphasize the necessity for antibiotics in conjunction with dental treatment only when there is clear evidence of systemic spread or a spreading superficial infection. Moreover, immediate, definitive and conservative dental treatment should always be prioritized [12,33].

The study found that the majority of respondents were aware of the appropriate use of antibiotics in high-risk medical conditions such as infective endocarditis, congenital cardiac abnormalities, and prosthetic cardiac valves. Antibiotic prophylaxis for infective endocarditis (IE) was first recommended by the American Heart Association (AHA) in 1955, with a long list of conditions that required prophylaxis, including native and prosthetic heart valve disease and pacemakers, among others [34]. However, current AHA guidelines only recommend IE prophylaxis for patients at higher risk for IE, such as those with a history of IE, certain types of congenital heart disease, and cardiac transplantation recipients with cardiac valvulopathy [35,36]. The current study was also consistent with the American Dental Association’s revised guideline, which advises against prophylactic antibiotics for dental procedures in patients with prosthetic joints, to which the majority of them correctly responded [37].

The majority of clinicians in the study preferred to treat their patients with amoxicillin, which aligns with the AHA guidelines [35]. Amoxicillin has a broad spectrum of action and has shown greater efficacy since it covers most bacteria responsible for oral infections. [38,39]. However, other studies have reported that amoxicillin with clavulanic acid is the first-choice antibiotic [19,26,40]. In Nigeria and India, amoxicillin and metronidazole are often prescribed together as the first [22,41] or second-choice antibiotic [42]. In the case of sensitivity to penicillin, the dentists in the current study identified clindamycin as the first drug of choice, followed by erythromycin, which is similar with other studies. However, the 2021 AHA scientific statement no longer recommends the use of clindamycin as an oral or parenteral alternative to amoxicillin or ampicillin in individuals with allergies to these drugs due to the possibility of more frequent and severe reactions, including C. difficile infection [35,43]. For patients with penicillin allergies, suggested alternatives now include first- or second-generation cephalosporins, azithromycin, clarithromycin, or doxycycline [35].

### 4.2. Knowledge scores

The majority of respondents in the current study showed high knowledge scores regarding antibiotic prescription, which is in contrast to some studies that produced contradictory results demonstrating various elements of medium to poor knowledge [19,27]. Interestingly, female respondents demonstrated significantly higher knowledge and practice scores than their male counterparts. This may be attributed to socialization patterns, as women are often encouraged to adhere to rules from an early age [44,45]. Additionally, studies have shown that women are more likely to follow evidence-based medicine guidelines [42,43].

In the present study, government-sector clinicians achieved the highest knowledge scores, followed by those in academia, while private-sector respondents scored comparatively lower; however, these differences were not significant. A study by Al-Huwayrini et al. [46] found that the majority of dentists working in private clinics in the Riyadh area had a good level of knowledge about prescribing antibiotics, whereas Baadani et al [47] concluded that both dentists in government and private practices in Saudi Arabia’s western region had good antimicrobial prescribing knowledge. A study conducted in the US showed that dentists significantly improved antibiotic prescribing patterns after enrolling in antibiotic stewardship education, including audits and weekly feedback from infectious disease experts which can be effectively implemented in Saudi Arabia [48].

There was a significant relationship between years of experience and knowledge of antibiotic prescriptions. Interestingly, those with less than five years of experience had more knowledge in this area compared to their more experienced counterparts. This finding was consistent with the work by Teoh et al., and Municki et al. [32,49] where the youngest respondents with the least clinical experience (one to five years) demonstrated the highest level of knowledge. The reason behind this trend is that these individuals have recently completed their formal university education and are therefore up-to-date with the latest guidelines. This suggests that other factors, such as changes in the curriculum and education system, may be influencing prescribing behaviour in addition to clinical experience.

### 4.3. Practice scores

The study’s results showed that two-thirds of the respondents had good antibiotic prescription practices. Clinicians in the government sector exhibited better performance in this area compared to private sector dentists. The majority of government clinicians were aware of antimicrobial resistance. As such, they were less likely to prescribe antibiotics without a verified diagnosis and a history of antibiotic use. In contrast, about 45% of private clinicians surveyed admitted that they would prescribe antibiotics to delay treatment in situations where there were long waiting lines. This finding is consistent with other studies conducted previously [50]. It is important to note that appropriate antibiotic use is crucial in combating antimicrobial resistance. Therefore, there should be continuous efforts to promote good antibiotic prescription practices among all clinicians, regardless of their sector.

The study utilized case-based scenarios, with over 50% of participants correctly identifying certain clinical cases. However, in other scenarios, less than half of the participants provided correct responses. These findings align with a study by Al-Johani K et al. [51], which used five clinical scenarios to evaluate dentists’ adherence to standards and reported overall adherence rates of less than 50% across all scenarios. This highlights significant gaps, including the limited clinical application of evidence-based knowledge, insufficient engagement from regulatory bodies in antibiotic prescription education, and inadequate data on dentists’ participation in training programs and prescription practices.

### 4.4. Limitations

A primary concern is the reliance on self-reported data, which is subject to inherent biases such as social desirability bias. Participants may over-report adherence to guidelines or under-report inappropriate prescribing practices, potentially inflating the perceived rates of guideline adherence. Additionally, the use of convenience sampling introduces the possibility of selection bias, limiting the ability to generalize findings across all dental practitioners in Saudi Arabia. The overrepresentation of government-sector dentists in the sample may have further skewed the findings, as these practitioners typically have better access to guidelines and training resources compared to their private-sector counterparts. This could lead to an overestimation of adherence rates.

### 4.5. Future recommendation

Based on the findings and limitations, several recommendations can be made. Regular antimicrobial stewardship programs tailored for dentists should be institutionalized to address knowledge gaps and promote rational antibiotic use. Dental councils must mandate participation in continuing professional development programs focused on antibiotic stewardship. Educational campaigns targeting antimicrobial resistance are essential to enhance awareness among practitioners. To improve future research, more objective methods such as anonymous patient record audits should be employed to minimize biases inherent in self-reported data. Additionally, adopting randomized or stratified sampling techniques would provide a more representative sample, ensuring balanced insights across public, private, and academic dental sectors. Regular evaluations of prescribing practices should also be conducted, with deliberate efforts to mitigate inappropriate antibiotic use in clinical settings.

## 5. Conclusion

Dentists in Kingdom of Saudi Arabia have a high level of expertise in antibiotics prescription. Individuals employed in the public sector, and academics were all substantially related with the proper pattern of antibiotic prescription. Additionally, amoxicillin and its derivatives are the most preferred drugs. However, some aspects of poor practices particularly among private sector clinicians necessitate the launching of educational campaigns, interventions and provision of latest guidelines for prudent use of antibiotics in dental practice.

## Supporting information

S1 File Inclusivity-in-global-research-questionnaire. (DOCX)

S1 Checklist Questionnaire annexure. (PDF)

## References

[1] Dar-Odeh N, Fadel HT, Abu-Hammad S, Abdeljawad R, Abu-Hammad OA. Antibiotic prescribing for oro-facial infections in the paediatric outpatient: a review. Antibiotics (Basel). 2018;7(2):38. doi: 10.3390/antibiotics7020038 29693642 PMC6022866

[2] Ramu C, Padmanabhan TV. Indications of antibiotic prophylaxis in dental practice- review. Asian Pac J Trop Biomed. 2012;2(9):749–54. doi: 10.1016/S2221-1691(12)60222-6 23570007 PMC3609373

[3] Marra F, George D, Chong M, Sutherland S, Patrick D. Antibiotic prescribing by dentists has increased: Why? J Am Dent Assoc. 1939;147(1):1–5. doi: 10.1016/j.adaj.2016.01.001 26857041

[4] Ahmadi H, Ebrahimi A, Ahmadi F. Antibiotic therapy in dentistry. Int J Dent. 2021;2021:6667624. doi: 10.1155/2021/6667624 33574843 PMC7861949

[5] Aslam B, Wang W, Arshad MI, Khurshid M, Muzammil S, Rasool MH, et al. Antibiotic resistance: a rundown of a global crisis. Infect Drug Resist. 2018;11:1645–58. doi: 10.2147/IDR.S173867 30349322 PMC6188119

[6] Löffler C, Böhmer F, Hornung A, Lang H, Burmeister U, Podbielski A, et al. Dental care resistance prevention and antibiotic prescribing modification-the cluster-randomised controlled DREAM trial. Implement Sci. 2014;9:27. doi: 10.1186/1748-5908-9-27 24559212 PMC3936853

[7] Antimicrobial Resistance Collaborators. Global burden of bacterial antimicrobial resistance in 2019: a systematic analysis. Lancet. 2022;399(10325):629–55. doi: 10.1016/S0140-6736(21)02724-0 35065702 PMC8841637

[8] D’Ambrosio F, Di Spirito F, Amato A, Caggiano M, Lo Giudice R, Martina S. Attitudes towards antibiotic prescription and antimicrobial resistance awareness among Italian dentists: What are the milestones? Healthcare (Basel). 2022;10(8):1585. doi: 10.3390/healthcare10081585 36011242 PMC9408165

[9] Lockhart PB, Hanson NB, Ristic H, Menezes AR, Baddour L. Acceptance among and impact on dental practitioners and patients of American Heart Association recommendations for antibiotic prophylaxis. J Am Dent Assoc. 2013;144(9):1030–5. doi: 10.14219/jada.archive.2013.0230 23989842

[10] Antimicrobial resistance. [cited 4 Sep 2024].https://www.who.int/news-room/fact-sheets/detail/antimicrobial-resistance

[11] FDI white paper: The essential role of the dental team in reducing antibiotic resistance | FDI. [cited 28 Dec 2024]. https://www.fdiworlddental.org/resource/fdi-white-paper-essential-role-dental-team-reducing-antibiotic-resistance

[12] Lockhart PB, Tampi MP, Abt E, Aminoshariae A, Durkin MJ, Fouad AF, et al. Evidence-based clinical practice guideline on antibiotic use for the urgent management of pulpal- and periapical-related dental pain and intraoral swelling: a report from the American Dental Association. J Am Dent Assoc. 2019;150(11):906–21.e12. doi: 10.1016/j.adaj.2019.08.020 31668170 PMC8270006

[13] Global action plan on antimicrobial resistance. [cited 28 Dec 2024]. https://www.who.int/publications/i/item/9789241509763

[14] Palmer N, Seoudi N, Ide M, Randall C, Hyland L, Patrick A. Antimicrobial Prescribing in Dentistry: Good Practice Guidelines. Royal College of Surgeons of England; 2020.

[15] Antibiotic Stewardship. [cited 28 Dec 2024]. https://www.ada.org/resources/ada-library/oral-health-topics/antibiotic-stewardship

[16] Al Marah Z, Abdulkareem A, Gul S, Alshami M. A survey of systemic antibiotic prescription patterns amongst Iraqi dentists. International Dental Journal. 2022;72(3):338–45. 34344542 10.1016/j.identj.2021.06.002 PMC9275136

[17] Chereau F, Opatowski L, Tourdjman M, Vong S. Risk assessment for antibiotic resistance in South East Asia. BMJ. 2017;358:j3393. doi: 10.1136/bmj.j3393 28874338 PMC5598255

[18] Talaat M, Zayed B, Tolba S, Abdou E, Gomaa M, Itani D. Increasing antimicrobial resistance in World Health Organization Eastern Mediterranean Region, 2017–2019. Emerging Infectious Diseases. 2022;28:717–24. 10.3201/eid2804.211975 PMC8962877 35318915

[19] Halboub E, Alzaili A, Quadri MFA, Al-Haroni M, Al-Obaida MI, Al-Hebshi NN. Antibiotic prescription knowledge of dentists in Kingdom of Saudi Arabia: an online, country-wide survey. J Contemp Dent Pract. 2016;17(3):198–204. doi: 10.5005/jp-journals-10024-1827 27207198

[20] Al Rasheed A, Yagoub U, Alkhashan H, Abdelhay O, Alawwad A, Al Aboud A, et al. Prevalence and predictors of self-medication with antibiotics in Al Wazarat Health Center, Riyadh City, KSA. Biomed Res Int. 2016;2016:3916874. doi: 10.1155/2016/3916874 26881218 PMC4736398

[21] Ab Rahman N, Teng CL, Sivasampu S. Antibiotic prescribing in public and private practice: a cross-sectional study in primary care clinics in Malaysia. BMC Infect Dis. 2016;16:208. doi: 10.1186/s12879-016-1530-2 27188538 PMC4869350

[22] Kumar KP, Kaushik M, Kumar PU, Reddy MS, Prashar N. Antibiotic prescribing habits of dental surgeons in hyderabad city, India, for pulpal and periapical pathologies: a survey. Adv Pharmacol Sci. 2013;2013:537385. doi: 10.1155/2013/537385 24187549 PMC3804281

[23] NICE - Antimicrobial Prescribing Guidelines | Department of Health. Health. 2018. [cited 4 Sep 2024]. https://www.health-ni.gov.uk/articles/nice-antimicrobial-prescribing-guidelines

[24] Germack M, Sedgley CM, Sabbah W, Whitten B. Antibiotic Use in 2016 by Members of the American Association of Endodontists: Report of a National Survey. J Endod. 2017;43(10):1615–22. doi: 10.1016/j.joen.2017.05.009 28754406

[25] Konde S, Jairam LS, Peethambar P, Noojady SR, Kumar NC. Antibiotic overusage and resistance: a cross-sectional survey among pediatric dentists. J Indian Soc Pedod Prev Dent. 2016;34(2):145–51. doi: 10.4103/0970-4388.180444 27080965

[26] Al-Harthi SE, Khan LM, Abed HH, Alkreathy HM, Ali AS. Appraisal of antimicrobial prescribing practices of governmental and non-governmental dentists for hospitals in the western region of Saudi Arabia. Saudi Med J. 2013;34(12):1262–9. 24343466

[27] Assery M, Al Khuzaei N, Al Rahbeni T, Al Mansoori M. Knowledge of antibiotics among dentists in Saudi Arabia. J Int Oral Health. 2017;9(2):71. doi: 10.4103/0976-7428.203634

[28] Alomran S, Alhosni A, Alzahrani K, Alamodi A, Alhazmi R. The reality of the Saudi health workforce during the next ten years 2018–2027. Saudi Comm Health Spec. 2017;1:17–9.

[29] Pai Khot AJ, Ankola AV, Sankeshwari RM, Choudhury AR, Kumar KRS, Shah MA. Knowledge, attitude, and practices toward tobacco control among rural community health care workers of primary subcenters in Belagavi district, Karnataka. J Family Med Prim Care. 2022;11(6):3257–69. doi: 10.4103/jfmpc.jfmpc_2216_21 36119189 PMC9480734

[30] Torumkuney D, Dolgum S, van Hasselt J, Abdullah W, Keles N. Country data on AMR in Saudi Arabia in the context of community-acquired respiratory tract infections: links between antibiotic susceptibility, local and international antibiotic prescribing guidelines, access to medicine and clinical outcome. J Antimicrob Chemother. 2022;77(Suppl_1):i70–6. doi: 10.1093/jac/dkac219 36065727 PMC9445845

[31] Cope AL, Francis NA, Wood F, Chestnutt IG. Antibiotic prescribing in UK general dental practice: a cross-sectional study. Community Dent Oral Epidemiol. 2016;44(2):145–53. doi: 10.1111/cdoe.12199 26507098

[32] Teoh L, Marino RJ, Stewart K, McCullough MJ. A survey of prescribing practices by general dentists in Australia. BMC Oral Health. 2019;19(1):193. doi: 10.1186/s12903-019-0882-6 31438922 PMC6704722

[33] Systemic antibiotics for symptomatic apical periodontitis and acute apical abscess in adults - PubMed. [cited 4 Sep 2024]. https://pubmed.ncbi.nlm.nih.gov/30259968/. 10.1002/14651858.CD010136.pub4 PMC11075121 38712714

[34] Prevention of rheumatic fever and bacterial endocarditis through control of streptococcal infections. Pediatrics. 1955;15:642–6. 14370902

[35] Wilson WR, Gewitz M, Lockhart PB, Bolger AF, DeSimone DC, Kazi DS, et al. Prevention of viridans group streptococcal infective endocarditis: a scientific statement from the American Heart Association. Circulation. 2021;143(20):e963–78. doi: 10.1161/CIR.0000000000000969 33853363

[36] Sperotto F, France K, Gobbo M, Bindakhil M, Pimolbutr K, Holmes H, et al. Antibiotic prophylaxis and infective endocarditis incidence following invasive dental procedures: a systematic review and meta-analysis. JAMA Cardiol. 2024;9(7):599–610. doi: 10.1001/jamacardio.2024.0873 38581643 PMC10999003

[37] Sollecito T, Abt E, Lockhart P, Truelove E, Paumier T, Tracy S, et al. The use of prophylactic antibiotics prior to dental procedures in patients with prosthetic joints: evidence-based clinical practice guideline for dental practitioners--a report of the American Dental Association Council on Scientific Affairs. J Am Dent Assoc. 2015;146(1):11–6.e8. 25569493 10.1016/j.adaj.2014.11.012

[38] Flynn TR. What are the antibiotics of choice for odontogenic infections, and how long should the treatment course last?. Oral Maxillofac Surg Clin North Am. 2011;23(4):519–36, v–vi. doi: 10.1016/j.coms.2011.07.005 21982604

[39] AAE Position Statement: AAE Guidance on the Use of Systemic Antibiotics in Endodontics. J Endod. 2017;43(9):1409–13. doi: 10.1016/j.joen.2017.08.015 28844223

[40] Iqbal A. The attitudes of dentists towards the prescription of antibiotics during endodontic treatment in North of Saudi Arabia. J Clin Diagn Res. 2015;9(5):ZC82-4. doi: 10.7860/JCDR/2015/13718.5964 26155570 PMC4484162

[41] Azodo CC, Ojehanon PI. Antibiotics prescription in Nigerian dental healthcare services. Odontostomatol Trop. 2014;37(147):34–42. 25975066

[42] Jayadev M, Karunakar P, Vishwanath B, Chinmayi SS, Siddhartha P, Chaitanya B. Knowledge and pattern of antibiotic and non narcotic analgesic prescription for pulpal and periapical pathologies- a survey among dentists. J Clin Diagn Res. 2014;8(7):ZC10-4. doi: 10.7860/JCDR/2014/9645.4536 25177628 PMC4149134

[43] Thornhill M, Dayer M, Prendergast B, Baddour L, Jones S, Lockhart P. Incidence and nature of adverse reactions to antibiotics used as endocarditis prophylaxis. J Antimicrob Chemother. 2015;70:2382–8. 25925595 10.1093/jac/dkv115 PMC4580535

[44] Howick J, Moscrop A, Mebius A, Fanshawe TR, Lewith G, Bishop FL, et al. Effects of empathic and positive communication in healthcare consultations: a systematic review and meta-analysis. J R Soc Med. 2018;111(7):240–52. doi: 10.1177/0141076818769477 29672201 PMC6047264

[45] Baumhäkel M, Müller U, Böhm M. Influence of gender of physicians and patients on guideline-recommended treatment of chronic heart failure in a cross-sectional study. Eur J Heart Fail. 2009;11(3):299–303. doi: 10.1093/eurjhf/hfn041 19158153 PMC2645055

[46] Al-Huwayrini L, Al-Furiji S, Al-Dhurgham R, Al-Shawaf M, Al-Muhaiza M. Knowledge of antibiotics among dentists in Riyadh private clinics. Saudi Dent J. 2013;25(3):119–24. doi: 10.1016/j.sdentj.2013.05.001 24179321 PMC3809504

[47] Baadani AM, Baig K, Alfahad WA, Aldalbahi S, Omrani AS. Physicians’ knowledge, perceptions, and attitudes toward antimicrobial prescribing in Riyadh, Saudi Arabia. Saudi Med J. 2015;36(5):613–9. doi: 10.15537/smj.2015.5.11726 25935184 PMC4436760

[48] Goff D, Mangino J, Trolli E, Scheetz R, Goff D. Private practice dentists improve antibiotic use after dental antibiotic stewardship education from infectious diseases experts. Open Forum Infectious Diseases. 2022;9:ofac361. 35959211 10.1093/ofid/ofac361 PMC9361170

[49] Šimundić Munitić M, Šutej I, Ćaćić N, Tadin A, Balić M, Bago I, et al. Knowledge and attitudes of Croatian Dentists Regarding Antibiotic Prescription in Endodontics: A Cross-sectional Questionnaire-based Study. Acta Stomatol Croat. 2021;55(4):346–58. doi: 10.15644/asc55/4/2 35001930 PMC8734455

[50] Karobari MI, Khijmatgar S, Bhandary R, Krishna Nayak US, Del Fabbro M, Horn R, et al. A multicultural demographic study to analyze antibiotic prescription practices and the need for continuing education in dentistry. Biomed Res Int. 2021;2021:5599724. doi: 10.1155/2021/5599724 34327231 PMC8310454

[51] Al-Johani K, Reddy S, Al Mushayt A, El-Housseiny A. Pattern of prescription of antibiotics among dental practitioners in Jeddah, KSA: a cross-sectional survey. Niger J Clin Pract. 2017;20:804–10. 28791973 10.4103/1119-3077.196072

