# Supplementary material for: Adherence to the current guidelines on antibiotic prescription among dental practitioners: A national survey
Source: PLoS One. 2025 Mar 31;20(3):e0320528. doi: 10.1371/journal.pone.0320528 (PMC11957268; doi:10.1371/journal.pone.0320528)
Supplement: S1 Checklist — (PDF) [file pone.0320528.s002.pdf]

Kingdom of Saudi Arabia
Ministry of Education
Princess Nourah bint Abdulrahman University
(048)
University Vice Rectorate for Graduate Studies and Scientific Research

المملكة العربية السعودية
وزارة التعليم
جامعة الأميرة نورة بنت عبدالرحمن
(٠٤٨)
وكالة الجامعة للدراسات العليا والبحث العلمي

Awareness of Latest Antibiotics Guidelines and Prescribing Practices of Saudi Dentists: National Survey

INFORMED CONSENT

Dear participants,

You are being asked to voluntarily participate in this survey research study. The purpose of the study is to understand the knowledge regarding antibiotics prescription and investigate the evidence-based practices among dental practitioners in Saudi Arabia. You are eligible to participate because you are a dentist working in Saudi Arabia. We expect at least 300 will participate in the survey in which is a online form.

If you agree to participate, your participation will involve completing a survey. It should take no more than 10 minutes. You may choose not to answer some or all of the questions. Your name will not appear on your completed survey, and no identifying information is being collected as part of this survey. Any questions you have will be answered. You may leave the survey at any time before completing it. Whether you complete the survey or not will not affect your health care. There are no known risks from your participation. No direct benefit from your participation is expected. The information may help understand the latest knowledge and practice regarding antibiotic use which helps to take necessary education programs for dentists. There is no cost to you except for your time. You will not be paid for participation in this study.

Only the study team will have access to the information that you provide, which will remain anonymous. Data from all respondents will be summarized in reports.
You can obtain further information from the principal investigator, Hoda M Abdellatif. If you have questions concerning your rights as a research subject, you may call the PNU Institutional Review Board office at 054 886 7916.

Completing this survey indicates your voluntary agreement to participate. By participating in the survey, you are giving permission for the investigator to use your information for research purposes. Finally, the researcher is personally liable for plagiarism and any violations of intellectual property rights.

Your participation is highly appreciated!

Thank you for your time and consideration.

Section A: Demographic data

**Gender**	☐ Female	☐ Male
**Age group (in years)**	☐ 25- 35<br>☐ 36- 45	☐ 46- 55<br>☐ > 55
**Clinical title**	☐ Consultant<br>☐ Specialist<br>☐ Resident<br>☐ General dentist<br>☐ Others, please specify ________	
**Practice sector**	☐ Clinics only (private sector)<br>☐ Clinics only (government sector)<br>☐ Academics only<br>☐ Clinics and Academics	
**Clinical experience**	☐ < 5 years<br>☐ 5-10<br>☐ 11-15<br>☐ > 15 years	
**Work place**	☐ Al- Jouf province<br>☐ Al-Bahah province<br>☐ Asir province<br>☐ Eastern province<br>☐ Hail province<br>☐ Jazan province<br>☐ Madinah province<br>☐ Makkah province<br>☐ Najran province<br>☐ Northern border<br>☐ Qassim province<br>☐ Riyadh province<br>☐ Tabuk province	
**Specialty**	☐ Endodontist<br>☐ Maxillofacial surgeon<br>☐ Pediatric dentist<br>☐ Periodontist<br>☐ General practitioner<br>☐ Others, please specify ________	
**Place of undergraduate training (select 1 or more)**	☐ Saudi Arabia<br>☐ Europe<br>☐ North America<br>☐ Other Asian countries<br>☐ Others, please specify _________	
**Place of postgraduate training (select 1 or more)**	☐ Saudi Arabia<br>☐ Europe<br>☐ North America<br>☐ Other Asian countries<br>☐ Others, please specify _________	

Section B: Knowledge of antibiotic prescription			
**1**= Yes **2** = No , **3** = Don't know	**1**	**2**	**3**
**1- Which of the following clinical conditions are indications for antibiotic prescription, in a medically fit patient?**			
Reversible pulpitis			
Irreversible pulpitis			
Pulp necrosis			
Apical periodontitis			
Draining dental sinus tract			
Localized intraoral abscess			
Cellulitis			
Acute ulcerative gingivitis			
Chronic marginal gingivitis			
Aggressive periodontitis			
Moderate periodontitis			
Mild pericoronitis			
Dry socket			
Fracture of tooth			
**2- Which of the following dental procedures are indications for antibiotic prescription, in a medically fit patient?**			
Scaling and Root planing			
Simple extraction			
Surgical extraction			
Root canal treatment			
Apicectomy			
Dental implant placement			
**3- Which of the following medical conditions are indications for antibiotic prophylaxis, before invasive dental procedures?**			
Cardiac pacemakers			
Congenital cardiac abnormalities			
Previous infective endocarditis			
Prosthetic cardiac valves			
Prosthetic joints			
**4- Which of the following dental procedures are indications for antibiotic prophylaxis, in a patient at risk for infective endocarditis?**			
Routine local anesthesia injections			
Scaling and root planing			
Extraction of teeth			

Restorative procedures			
Endodontic procedures			

Section C: Antibiotic prescription practice

**1- Do you prescribe antibiotics in these situations?**	Always	Never	Occasionally
If clinical condition demands antibiotic prescription			
If patient requests antibiotic prescription			
If you are uncertain of diagnosis			
To sustain patients, until specialist is available			
To defer the treatment, in case of long waiting queues			

2- Are you aware of "antimicrobial resistance"?

- ☐ Yes
- ☐ No

3- Do you inquire from your patient about whether he/she has taken a course of antibiotics in the past 1 week, before prescribing antibiotics?

- ☐ Yes
- ☐ No
- ☐ Occasionally

4- Do you advise your patient to adhere to the dosage regimen and inform the consequences of not doing so?

- ☐ Yes
- ☐ No
- ☐ Occasionally

5- Which antibiotic(s) do you most often prescribe therapeutically, for your patients? (pop-up question for the selected antibiotic to enquire about dose, frequency, duration)

- ☐ Amoxicillin
- ☐ Amoxicillin & Clavulanic acid
- ☐ Amoxicillin & Metronidazole
- ☐ Cephalosporin/Cephalexin
- ☐ Clindamycin
- ☐ Azithromycin
- ☐ Ciprofloxacin
- ☐ Ofloxacin
- ☐ Metronidazole
- ☐ Ornidazole
- ☐ Others, please specify __________

6- Which antibiotic do you prescribe for your patients, allergic to penicillin?

- ☐ Erythromycin
- ☐ Azithromycin
- ☐ Clindamycin
- ☐ Clarithromycin
- ☐ Cephalosporin
- ☐ Others, please specify __________

7- Do you follow current guidelines for antibiotic prophylaxis?

- ☐ Always
- ☐ Never

☐ Occasionally

If yes, which guidelines do you follow?

☐ AHA guidelines
☐ NICE guidelines
☐ Others, please specify______

8- What was your main source of updating your knowledge regarding antibiotic prescription guidelines?

☐ Textbooks
☐ Conferences
☐ Journal articles
☐ Continuing education courses
☐ Colleagues
☐ Others, please specify __________

9- When did you last update your knowledge regarding antibiotic prescription guidelines?

☐ Within the last two years
☐ Within the last five years
☐ Others, please specify __________

10- Do you prescribe antibiotics in the following clinical situations?

**1=** Yes **2 =** No, **3 =** Don't know	**1**	**2**	**3**
You have just performed a surgical extraction of grossly decayed #46, in a known diabetic patient whose random blood glucose level was 180 mg/dl. The surgery lasted about one hour long.			
In ER, a parent reported that one of her 9-year old child's teeth was extremely painful. The child was afebrile. However, an intraoral swelling was found in relation to #74, along with halitosis and discharging pus.			
A 6-year old girl reported to the ER with a history of fall, while skating on the road. On clinical examination, her upper lip was edematous and the anterior maxillary alveolar segment was mobile.			
You have planned to perform scaling and root planing in a 20-year old male patient who was already diagnosed with a genetic disorder, Marfan syndrome who had undergone repair of mitral valve 2 years ago.			
A 23-year old patient presented with bad breath, restricted mouth opening and pain in relation to the partially erupted lower third molar. The area around the tooth was erythematous and swollen.			
You noticed a vertical fracture in a painful right upper first molar extending to the furcation, and made a treatment plan of extraction. However, the patient is unwilling to undergo extraction.			
A 65-year old patient with chronic kidney disease, undergoing hemodialysis twice every week needs an endodontic treatment of #46, Tooth is associated with a periapical abscess.			
You advise a female patient to come for periodontal surgery after two days. Her medical history is unremarkable, except that she is a carrier of sickle cell anemia.			
You need to perform incision and drainage of a submandibular swelling in a patient with a history of coronary artery bypass grafting six months ago. Her general physical status is currently stable.			

An apprehensive parent, reports that her daughter had multiple ulcers of lower lip from the time orthodontic bonding done a week ago, as they would be out of town for ten days, she insists for an antibiotic prescription for her daughter to prevent infection.			
